# Combining video telemetry and wearable MEG for naturalistic imaging

**DOI:** 10.1162/imag_a_00495

**Published:** 2025-03-03

**Authors:** George C. O’Neill, Robert A. Seymour, Stephanie Mellor, Nicholas A. Alexander, Tim M. Tierney, Léa Bernachot, Mansoureh Fahimi Hnazaee, Meaghan E. Spedden, Ryan C. Timms, Daniel Bush, Sven Bestmann, Matthew J. Brookes, Gareth R. Barnes

**Affiliations:** Department of Neuroscience, Physiology and Pharmacology, University College London, London, United Kingdom; Department of Imaging Neuroscience, UCL Queen Square Institute of Neurology, University College London, London, United Kingdom; Oxford Centre for Human Brain Activity (OHBA), Department of Psychiatry, University of Oxford, Oxford, United Kingdom; Spinal Cord Injury Center, Balgrist University Hospital, University of Zurich, Zurich, Switzerland; Translational Neuromodeling Unit (TNU), Institute for Biomedical Engineering, University of Zurich & ETH Zurich, Zurich, Switzerland; Institute of Cognitive Neuroscience, University College London, London, United Kingdom; Department of Clinical and Movement Neurosciences, UCL Queen Square Institute of Neurology, University College London, London, United Kingdom; Sir Peter Mansfield Imaging Centre, School of Physics and Astronomy, University of Nottingham, Nottingham, United Kingdom

**Keywords:** OPM, MEG, naturalistic neuroscience, telemetry, pose estimation

## Abstract

Neuroimaging studies have typically relied on rigorously controlled experimental paradigms to probe cognition, in which movement is restricted, primitive, an afterthought or merely used to indicate a subject’s choice. Whilst powerful, these paradigms do not often resemble how we behave in everyday life, so a new generation of ecologically valid experiments are being developed. Magnetoencephalography (MEG) measures neural activity by sensing extracranial magnetic fields. It has recently been transformed from a large, static imaging modality to a wearable method where participants can move freely. This makes wearable MEG systems a prime candidate for naturalistic experiments going forward. However, these experiments will also require novel methods to capture and integrate information about behaviour executed during neuroimaging, and it is not yet clear how this could be achieved. Here, we use video recordings of multi-limb dance moves, processed with open-source machine learning methods, to automatically identify time windows of interest in concurrent, wearable MEG data. In a first step, we compare a traditional, block-designed analysis of limb movements, where the times of interest are based on stimulus presentation, to an analysis pipeline based on hidden Markov model states derived from the video telemetry. Next, we show that it is possible to identify discrete modes of neuronal activity related to specific limbs and body posture by processing the participants’ choreographed movement in a dancing paradigm. This demonstrates the potential of combining video telemetry with mobile magnetoencephalography and other legacy imaging methods for future studies of complex and naturalistic behaviours.

## 1 Introduction

One of the challenges for neuroimaging is the development of more*naturalistic*and ecologically valid approaches for studying brain function (Finn et al., 2022;Sonkusare et al., 2019). Much effort has been made in creating complex visual stimuli to assess brain function in a way which is closer to the real world than the laboratory (Betti et al., 2013;Kim et al., 2018;Kringelbach et al., 2023). However, a fundamental limitation remains in that the actual expression of behaviour through movement is limited in most neuroimaging modalities. For example, it was recently demonstrated that patients with temporal lobe resections struggle to navigate a virtual environment when exploring it from a desktop computer, but navigate as well as healthy controls when exploring in an immersive virtual reality environment (Iggena et al., 2023). The restrictive nature of most neuroimaging studies means we can isolate certain functions of the brain, but lose understanding of how they integrate as a part of a wider network, ultimately limiting the ecological validity of studies of brain function. To this end, mobile neuroimaging modalities, such as functional Near Infra-Red Spectroscopy (fNIRS) and electroencephalography (EEG), have been adopted for immersive, ambulatory, naturalistic studies (Aghajan et al., 2017;Alexander et al., 2024;Burgess et al., 2022;Gehrke and Gramann, 2021;Oliver et al., 2018;Richer et al., 2024;Töllner et al., 2017).

Magnetoencephalography (MEG;Cohen, 1972;Hämäläinen et al., 1993) is a powerful non-invasive method of imaging neural function from the brain, which measures the changes in extracranial magnetic field originating from intra-cellular current flow. As MEG is a direct measure of underlying neural processes, it offers a millisecond-scale temporal resolution to capture the rapid dynamics of cognition. Unlike EEG which measures the same signal via electric potentials on the scalp, magnetic fields pass through the skull with relatively little distortion, allowing MEG to offer a higher spatial resolution when the data are source-reconstructed. However, magnetic fields from neural populations in the brain are of the order of tens of femtoTesla, so traditional MEG systems have required an array of highly sensitive superconducting sensors and cumbersome cryogenic infrastructure to support them. This means that participants have to keep their head (and by extension, the rest of their body) as still as possible within a gantry which contains the sensing array. While some attempts at naturalistic MEG studies have been made (e.g., listening to speech or watching movies;Betti et al., 2013;Nunes et al., 2020;Park et al., 2016;Thiede et al., 2020), the constraint on movement makes these studies passive, with participants not behaving in a natural way during the presentation of the stimulus.

Recent advances in the engineering of cryogen-free sensors, particularly optically pumped magnetometers (OPMs;Knappe et al., 2016;Osborne et al., 2018;Shah and Wakai, 2013;Sheng et al., 2017), have led to the rapid development of a new generation of optically pumped (OP) MEG systems (Borna et al., 2017;Boto et al., 2017;Iivanainen et al., 2019;Pratt et al., 2021). OP-MEG has been used to assess sensory (Hill et al., 2019;Rier et al., 2024;Roberts et al., 2019;Seymour et al., 2021) and cognitive processes (Barry et al., 2019;de Lange et al., 2021;Rhodes et al., 2023;Tierney et al., 2018), as well as showing its potential for clinical applications (Feys et al., 2022,^2023^;Hillebrand et al., 2023;Vivekananda et al., 2020). The diminutive size of commercial OPMs (approximately the size of a 2-by-3 LEGO brick) means that many of these sensors can be packed into a rigid helmet (Boto et al., 2017) or soft cap (Feys et al., 2022;Hill et al., 2020), close to the scalp and worn in a form factor similar to EEG. A further advantage is the fact that muscle activity from the neck or other body parts does not contaminate the MEG signal to the same proportion as in EEG (Boto et al., 2019;Muthukumaraswamy, 2013), which can prove highly beneficial if a study requires explicit movement. Indeed, OP-MEG has already demonstrated compatibility with immersive virtual reality environments (Roberts et al., 2019) and has been used to reliably image neural processes while participants move large (>1 m) distances (Holmes et al., 2023b;Mellor et al., 2023;Seymour et al., 2021), play a ball-game against each other (Holmes et al., 2023a), or simply drink tea (Boto et al., 2018). It is for these reasons that OP-MEG promises to be a powerful tool in the field of naturalistic imaging with unconstrained movement (Stangl et al., 2023).

One challenge to this approach, however, is how to incorporate complex behaviour into the modelling of neuroimaging data as experiments become increasingly realistic (Stangl et al., 2023). Recent developments in computer vision and machine-learning methods to track behaviour offer a promising approach (Anderson and Perona, 2014;Bigand et al., 2024;Kaneko et al., 2024;Mathis et al., 2018;Schneider et al., 2023;Weinreb et al., 2023). Here, we demonstrate how behavioural information that is extracted from a data-mined video of participants performing motor paradigms can be fused with concurrently recorded wearable MEG data to yield brain measures epoched and extracted from behaviour. We first show that we can decode the experimental state of a participant from the video data alone. Next, we show that by using the video, we can return context to MEG data from participants dancing and use the video-derived cues to quantify neuronal activity associated with the movement of specific limbs. In sum, we describe a largely automated method of processing video data to identify time windows of interest in concurrently recorded neuroimaging data, thereby providing an analysis pipeline to support future naturalistic experiments across a range of cognitive domains.

## 2 Methods

### 2.1 Experiment

This study was carried out at the OP-MEG scanning suite at the Department of Imaging Neuroscience, UCL. The project was approved by the University College London research ethics committee. All participants who took part in the study provided informed written consent prior to MEG/MRI data collection.

#### 2.1.1 Motor block design

Three participants (all male, aged 43 ± 12 [mean ± SD years]) took part in a motor paradigm. Seated in the middle of the magnetically shielded room (MSR), the participant was visually cued to move one of their four limbs freely until a fixation cross appeared on the screen. The movement epochs were 4 s in length with a 10–11 s inter-trial interval. In a block, 15 trials of each condition were presented in a pseudorandom order, with two blocks recorded per participant.

#### 2.1.2 Dancing

Five participants (all male, aged 37 ± 12 [mean ± SD years]) danced the*Hokey Cokey*, a popular dance for school children in the United Kingdom. It was selected as its major actions involve moving individual limbs separately, with periods where the lyrics explicitly instruct the dancer how to move (“you put your left arm in…”) as well as periods of ambiguity (“whoa, the Hokey Cokey!”). Participants were given basic choreography training prior to recording. An audio recording of the song (Black Lace, 1985) was played into the room from a set of speakers placed outside of the room. Each dance lasted 158 s, and participants repeated the dance multiple times. In total, 20 recorded dances across all participants were kept for further analysis.

### 2.2 Acquisition

#### 2.2.1 Magnetic resonance imaging

Each participant underwent Magnetic Resonance Imaging (MRI) in preparation for the study on a Tim Trio 3T MR System (Siemens Healthineers, Erlangen, Germany). Two images were acquired for each participant. The first was a modified FLASH sequence with a high-bandwidth readout (FOV: 256 mm (A-P) x 256 mm (S-I) x 192 mm (L-R); resolution 1 mm x 1 mm x 1 mm) to minimise distortion of the participants’ face and scalp, while maintaining enough dynamic range to segment white and grey matter in the brain. Full details of the acquisition parameters can be found inMeyer et al. (2017). The second, a T1-weighted image (MPRAGE; TR = 2530 ms, TE = 3.34 ms; FOV: 256 mm (A-P) x 256 mm (S-I) x 172 mm (L-R); resolution 1 mm x 1 mm x 1 mm) was collected to supplement the first scan, in case automatic segmentation methods with the FLASH MR image failed.

#### 2.2.2 Meg

The OPM arrays consisted of a combination of dual axis (2^nd^generation) and triaxial (3^rd^generation) zero-field magnetometers (QuSpin, Louisville, CO): the dual axis sensors provided axially-oriented field detection and one tangential field measure; the triaxials gave a full vector field measurement. The sensors operated in an open-loop mode with an operational dynamic range of ~ ± 4.5 nT relative to their zero-field point. The number of channels recorded ranged from 64 to 128; detailed breakdowns of channel counts and sensor layouts are available in theSupplementary Material. The MEG data were digitised using a 16-bit ADC system (National Instruments, Austin, TX) at a sample rate of 6000 Hz.

The sensors were placed in bespoke 3D-printed scanner-casts (Chalk Design, London, UK) specifically designed for each participant (Boto et al., 2017;Tierney et al., 2018). Scanner-casts ensure a comfortable fit and minimise the co-registration errors between the sensors and the participant’s anatomy (Meyer et al., 2017). The helmet’s geometry was based on a scalp mesh extracted from the FLASH MR images. The OPMs were oriented such that the manufacturer-defined Y-axis measured the component of the magnetic field axial/radial to the scalp, with the centre of the vapour cell typically between 9 and 12 mm from the scalp surface. Sensor locations were in the same coordinate system as the anatomical images due to the manufacturing process of the scanner-casts, so no additional registration was required.

MEG data were acquired in an MSR (Magnetic Shields Ltd, Staplehurst, UK), with internal dimensions of 4380 mm x 3380 mm x 2180 mm. The MSR is constructed from two inner layers of 1 mm mu-metal, a 6 mm copper layer, and then two external layers of 1.5 mm mu-metal. The room contains a series of built-in degaussing coils to minimise the residual background field in the room (Altarev et al., 2015). The degausser was used in the period after closing the participant in the room and prior to data acquisition. No external active magnetic shielding was used for these experiments.

#### 2.2.3 Video

Visible-light spectrum video recordings of the participants performing the experiments were recorded alongside the MEG data using a camera attached to a single-board computer (Raspberry Pi Foundation, Cambridge, UK). The camera was triggered to record via a GPIO pin controlled by the stimulus presentation software, sent to both the camera and the OPM acquisition electronics to allow for offline synchronisation. Video was recorded at a resolution of 640 x 480 pixels with a frame rate of 30 Hz (with the exception of subjects 004 and 005, which were recorded at 50 Hz). The camera was located in front of participants 001, 002, and 005 (seeFig. 1for an example), and approximately 45 degrees off axis to the right of participants 003 and 004.

**Fig. 1.**
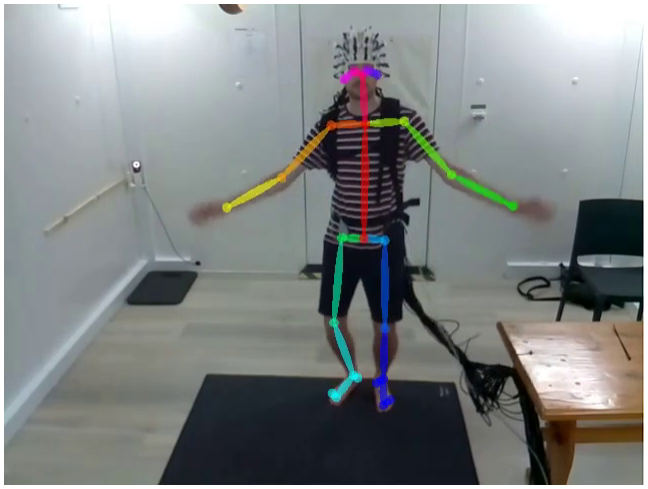
A subject performing the dancing paradigm within the magnetically shield room while wearing the OP-MEG system. The pose estimation results for this video frame have been superimposed to represent where the key-points (circles) used for telemetry analysis were located.

### 2.3 Preprocessing

#### 2.3.1 Video

Telemetry information from the video was extracted using OpenPose (Cao et al., 2021), a pre-trained convolutional neural network which is capable of single- and multi-person pose estimation from images and video. Inference was performed using an RTX A5000 GPU (NVIDIA, Santa Clara, CA), and for every frame, the X-Y pixel locations and a confidence-of-fit score for 25 different key-points of the body were recorded. An example of the key-points fitted to each frame can be found inFigure 1. For each experiment, the telemetry position data were linearly interpolated to fix missing data, converted to speed (units: pixels / frame), and normalised via a Z-transform. Finally, the 50 Hz telemetry from participants 004 and 005 was linearly interpolated to match the 30 Hz video sampling rate of subjects 001–003. For the dancing telemetry, each session’s data were normalised individually and then concatenated in time. The data were then partitioned using a hidden Markov model (HMM), with a multivariate Gaussian mixture model used as the observation model. To derive when differing movement ‘states’ occurred in the data, HMM inference was handled with the HMM-MAR toolbox (Vidaurre et al., 2016) to separate the data intokstates. On completion, the fitted HMM returned a probabilistic timeseries for each of thekstates and Viterbi path which assigns a mutually-exclusive state on a per-frame basis.

In the block design experiment, we setk=15. To identify what each state corresponded to, the binarised Viterbi path for a given state was compared to the initial block design timings using the Jaccard index. The Jaccard index is a measure of the intersection of two sets relative to their union (Jaccard, 1912). The state with the highest Jaccard index for a given experimental condition was labelled with that condition name. The 11 remaining (unlabelled) states were considered as rest and combined into a single rest*meta-state*.

For the dancing experiment, where the video consisted of a concatenated group of subjects (from different angles in the room) we fitted a larger,k=25state HMM. The higher number of requested states would allow for multiple states corresponding to the same limb (but from different subjects) to be identified. After HMM fitting, the Viterbi paths were regressed against the telemetry of each key-point, to generate regression maps of movement (examples can be seen inFigs. 2Band4A). From these maps, which limb (or dance move) a state corresponded to was performed by visual inspection, with appropriate labelling of the states applied. If two or more states represented the same limb (but across different subjects), their Viterbi paths were pooled together to make one meta-state representing the limb across all subjects (details of state-limb mappings can be found in theSupplementary Material).

**Fig. 2.**
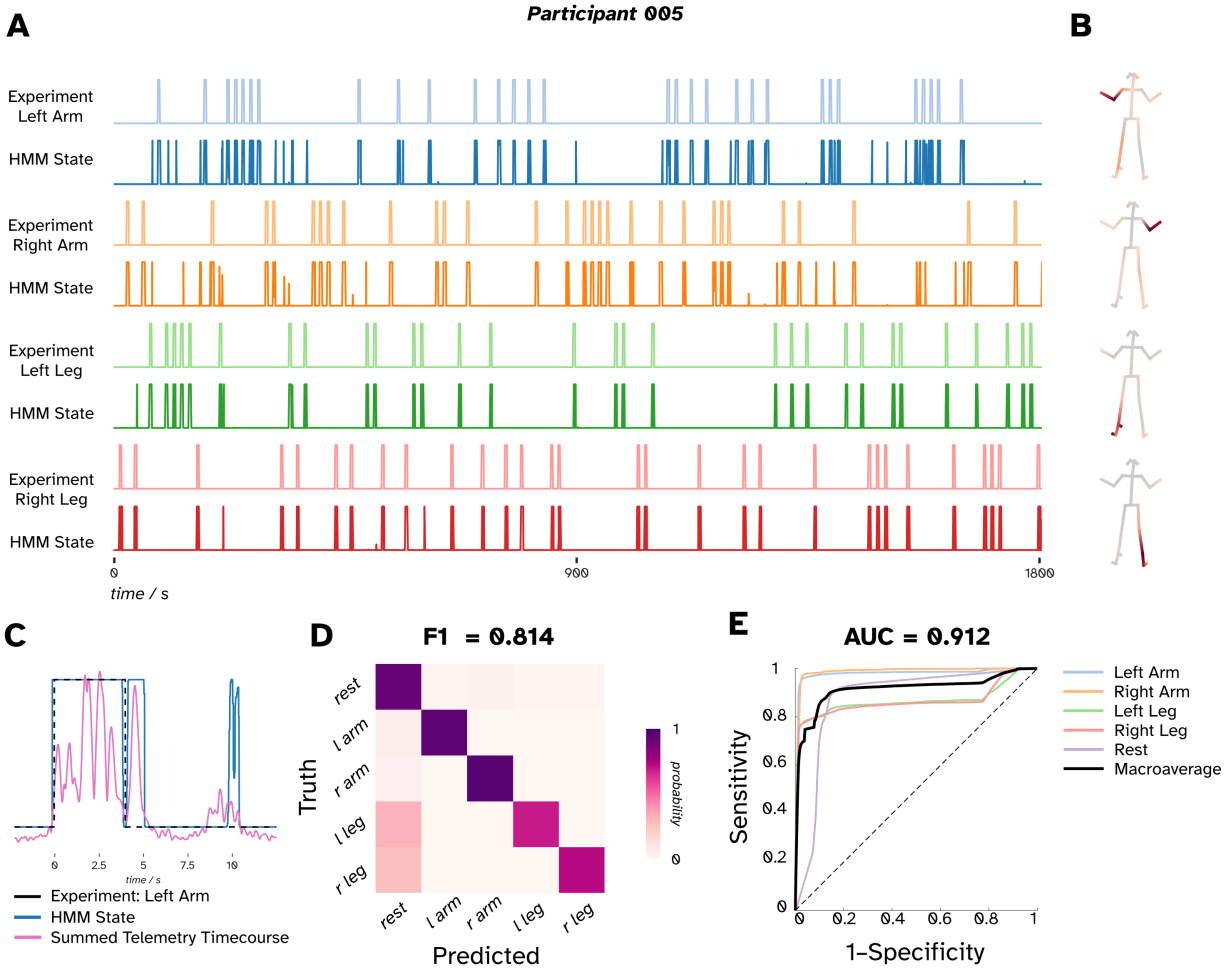
A comparison of the HMM-derived video telemetry states in comparison to the original experiment design for a simple 4 condition motor task in a single subject (participant 005). (A) Temporal plots depicting the 4 experimentally derived states across a recording represented as binary signals. Plotted below each experimental timecourse is the corresponding telemetry state that most closely matched each experimental condition. The duration of each movement was 4.2 ± 0.9 s (mean ± SD). (B) Heatmaps corresponding to the regression of the HMM state timecourse against the original telemetry data to identify which body parts were moving in a given state; the darker the plot, the stronger the relationship between telemetry data and a given HMM state. (C) A portion of the left arm experimental state (black dashed line) and the closest matched HMM state (blue solid line) which demonstrates the delay (~1000 ms offset) between presentation of experimental cue and cessation of movement. We also see a period of small movements (pink line) occurring at around 8 s (during the inter-trial interval) which was classified as movement by the HMM, (D) Multi-class confusion matrices showing the probability of a given HMM state being active compared to the experimental ground truth. (E) Receiver operating characteristic (ROC) curves for the resultant HMM compared to the block design it is predicting. Pale colours represent the states representing specific conditions in a (one vs. all) classification. The black line represents the macro-average (unweighted mean) of all 5 ROC curves.

#### 2.3.2 Meg

An anti-aliasing 500 Hz low-pass filter (60th-order FIR filter combined with a Kaiser window) was applied to the MEG data and then downsampled to a sample rate of 2000 Hz. The HMM state time courses derived from the video were synchronised to the MEG using nearest-neighbour interpolation and appended to the dataset as supplementary trigger channels. Environmental interference was reduced by applying a Homogenous Field Correction (HFC), where the interference is modelled as a set of regular spherical harmonics derived from the sensor array (Tierney et al., 2022). Here, 8 components were projected out of the data (3 homogenous field components and 5 linear gradients). If any channels had a residual variance larger than 100 pT after HFC, the projection was undone, these channels were disabled, and the HFC was reapplied again without those channels included in the model. The sensor compensation matrix was also updated to account for the linear combinations of sensors in the forward modelling (Tierney et al., 2021). A secondary advantage of using HFC is that its performance is robust across arrays of differing channel numbers and types. The expected difference in signal is <1 dB across the arrays utilised (Tierney et al., 2022). We then band-pass-filtered into the 8–30 Hz band, which has been strongly associated with sensorimotor activity (Gaetz et al., 2020;Jurkiewicz et al., 2006;Pfurtscheller & Lopes da Silva, 1999), though we note that these are often separated into sub bands with distinct purposes (Richer et al., 2024).

### 2.4 Source localisation

Source localisation was performed with the DAiSS toolbox supplied with SPM12 (Litvak et al., 2011). Sources were modelled along a 5 mm grid within a boundary delineating the brain and cerebrospinal fluid (CSF) of the participant. The forward model used was Nolte’s single shell (Nolte, 2003), where the conductive volume geometry was the same brain/CSF boundary. Three dipoles per location representing the cardinal orientations were generated, with their degrees of freedom reduced to two to compensate for the ‘silent radial source’. A linearly-constrained-minimum-variance (LCMV) beamformer (Brookes et al., 2008;Van Veen et al., 1997) was implemented to perform the inverse modelling. Due to the projection carried out during HFC in preprocessing, MEG data were rank deficient. To control for this, the covariance matrix was regularised; the matrix was decomposed into a set of eigenvectors and eigenvalues, and the 8 smallest eigenvectors/eigenvalues (associated with the 8 projected-out components from HFC) were discarded (Westner et al., 2022). During LCMV, the forward models for each source were linearly combined to maximise variance from that location (Sekihara et al., 2004).

### 2.5 First-level source analysis

To map which sources in the brain covaried with the onset and offset of the behavioural states, we opted to take a 2-level general linear model (GLM) approach (Brookes et al., 2004;Quinn et al., 2024;Worsley & Friston, 1995). In particular, we took an approach based on the GLM-beamfomer (Brookes et al., 2004). For the first level, we performed a mass-univariate multiple regression on each source. For each source we generated an amplitude envelope timecourse via a Hilbert transform. Prior to regression, these envelopes were log-transformed (Hawkins & Wixley, 1986). A given processed source signal$y\in {\mathbb{R}}^{n_{samples}\times 1}$is fitted to our general linear model:

y=Xβ+e

where,$X\in {\mathbb{R}}^{n_{samples}\times n_{regressors}}$, is our design matrix with our temporal features of interest in the columns,$\beta \in {\mathbb{R}}^{n_{regressors}\times 1}$are the regression coefficients, and$e\in {\mathbb{R}}^{n_{samples}\times 1}$are our unexplained data. How we constructedXfor each experiment differed as follows. For the motor experiment, we generated two design matrices which differed in how the first column was defined. Column one was either the active period as specified by the block design (a box car between 0 and 4 s of trial onset), or the HMM-derived active period (binarised state time-series). For both design matrices, the second column was a baseline ‘rest’ period (box car between 6 and 10 s of trial onset) and the third column modelled was the mean. A regression was performed for each source in the brain, generating a set of regression coefficients per voxel, which were combined to generate images. For each trial, two regression coefficient images (active and rest features) were kept for further analysis.

For the dancing experiment design, we included all motor meta-states as regressors as well as a mean and linear trend features, as our motor states were orthogonal in time. A single design matrix per dance was constructed to generate one regression coefficient image per meta-state, per dance. Example design matrices for both experiments can be found in theSupplementary Material.

### 2.6 Second-level analysis

#### 2.6.1 Motor block design

In MEG studies, it is typical to contrast the movement period to a baseline (rest) epoch within the same trial (e.g.,Pakenham et al., 2020). We replicate this approach in our second-level GLMs. For each limb, the 1st-level regression coefficient images (either block design or HMM timed) were put into a paired t-test design matrix with their trial-specific rest regression coefficient images and a t-contrast between the active and rest images were generated, forming a paired t-image for each limb.

#### 2.6.2 Dancing

With no obvious hypothesis as to where a ‘rest’ period would occur in the data, we opted to contrast different movement states to each other (Ma et al., 2022). Regression coefficient images from each dance were first put into a 1 x 5 factorial design matrix, and t-contrasts between various limbs were generated. We opted to treat each dance individually. For all t-images, a whole-brain Family-Wise Error (FWE) correction using the volumetric random field theory (Worsley et al., 1996) was applied using SPM.

## 3 Results

### 3.1 Motor block design

We set out to assess whether in a controlled, block-designed motor paradigm we could detect participant movement in video telemetry and use that to localise movement-related activity in the brain.Figure 2depicts the results from a typical participant decoding the telemetry of the block-design experiment, where 1 of the 4 limbs were moved during a visual cue period. Similar results from the other participants can be found in theSupplementary Material.Figure 2Ashows the binary timecourse for each condition of the experiment and the closest matching HMM state timecourse. Here, the metric for matching a state to the original timecourse was the Jaccard index (mean ± SD) for the selected 4 states of 0.65 ± 0.06 (p < 0.001, seeSupplementary Material). The resultant HMM states qualitatively resemble the original experimental timeseries they were compared against. The regression heatmaps inFigure 2Bshow which key-points on the body were implicated in each state. We observe that the parts of the body the regression highlighted correspond to the pre-defined experimental condition.

One feature that the telemetry identifies is the reaction time between a stimulus being presented and the participant executing and concluding the movement.Figure 2Cillustrates this with an example trial (where the participant was asked to moved their left arm) in which the black dashed line represents the stimulus timing, and the blue solid line corresponds to the posterior probability of the state being active. Here, we observe that the participant took (approximately) an extra second to cease movement after they were cued to stop. We found that across all trials, the total time movement was executed for was 4.2 ± 0.9 s (mean ± SD).Figure 2Calso contains an additional period where the state being active is highly active at around t = 10 s, which was within the inter-trial interval. To illustrate why the state is active, we have overlaid the associated telemetry timeseries from that state (made of the weighted sum of the key-points highlighted inFig. 2B). We see the HMM is also sensitive to spurious smaller movements after the (instructed) large movements during the trial. These two examples withinFigure 2Chighlight how the telemetry data can reveal when the participant deviates from the experimental design.

We quantitatively compare the decoding of the experiment to the prescribed experimental timings inFigure 2D–E. These should be interpreted in the context of the variable reaction times of the participants.Figure 2Dis the confusion/classification matrix comparing the ground truth of the experimental condition to the HMM-derived Viterbi path. The Viterbi path is binarised for each labelled experimental state. Note for the ‘rest’ state, this represents the remaining 11 unlabelled states from the HMM. For each condition, the predicted state was the most dominant, and we get an overall F_1_score (non-weighted average of the Dice coefficients;Manning et al., 2008) of 0.814 (p < 0.001, seeSupplementary Material). For the movement conditions, we see that a proportion of the movement states are predicted to be the rest condition, which would correspond to the reaction time for the participant to initiate the movement after the cue.Figure 2Eshows the Receiver Operatic Characteristic (ROC) curves for the HMMs probabilistic state timeseries. Again, the timeseries for the unlabelled states are pooled together to represent the ‘rest’ condition. Here, we are assuming a one-versus-all classification (i.e., correctly classifying a given state vs. not). The areas under the curve (AUC) for each of the states are high (left leg, 0.868; right leg, 0.862; left arm, 0.975; right arm, 0.990; rest, 0.8684), resulting in a macro-average (non-weighted average) AUC of 0.912 (p < 0.001, seeSupplementary Material). We also performed a split-half cross-validation, which showed similar levels of performance (seeSupplementary Material).

**Fig. 3.**
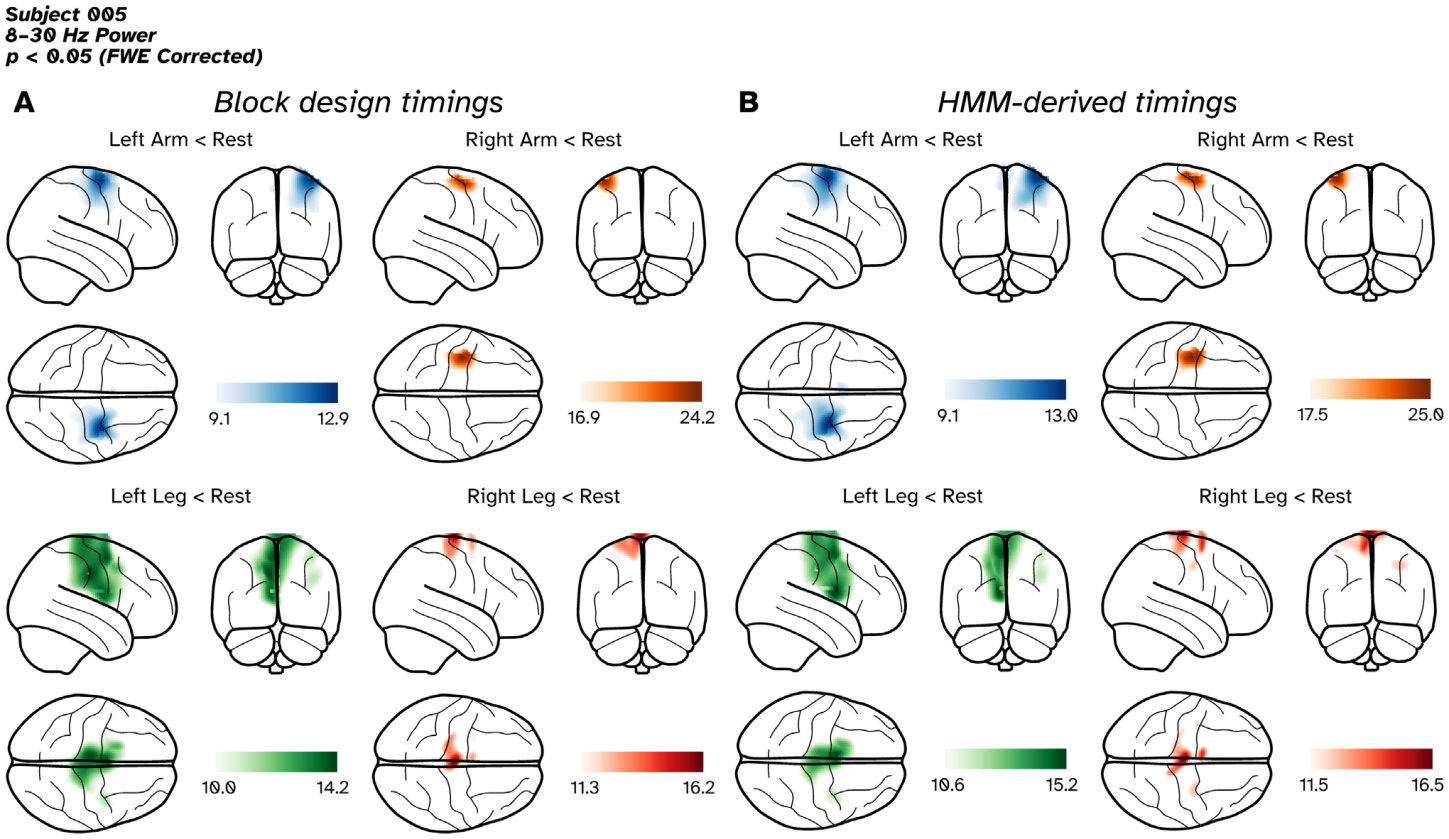
Source localisation results of a block-designed, 4-condition motor experiment in a single subject, with results presented on a glass brain. (A) T-contrasts for the 4 active conditions versus rest based on the timings specified by the block design. (B) T-contrasts for the 4 active conditions versus rest based on the HMM-derived timings. Images have been thresholded to show anything within 70% of the most extreme statistic.

Figure 3shows the T-contrasts where 8–30 Hz oscillatory power in rest periods were significantly higher (p < 0.05, FWE corrected) than the original experimental-derived active epochs (Fig. 3A) or the HMM-derived epochs for each of the 4 experimental conditions (Fig. 3B). For clarity, we have applied a threshold of 70% of the most extreme T-statistic in the image. In all conditions, we observe the characteristic event-related decrease in power during movement. Focusing on the block-design derived results (Fig. 3A), we first observe that all of the peak locations are in the precentral gyrus (motor cortex). Second, we see that all 4 of the images follow the traditional organisation of the motor cortex (Gordon et al., 2023), with the arm condition peaks localised more laterally, and the leg peaks appearing medially. The peak T-value locations can be found in theSupplementary Material. Considering the results of the HMM-derived timings, we note two things. First, the peak locations are very close between the two approaches (distances between peaks no further than 9.2 mm apart, seeSupplementary Material). Second, the threshold statistical maps are similar in their morphology and their T-statistics are of a similar magnitude to the block design images. To summarise, the telemetry-based analysis gives comparable results to the block design.

### 3.2 Dancing

**Fig. 4.**
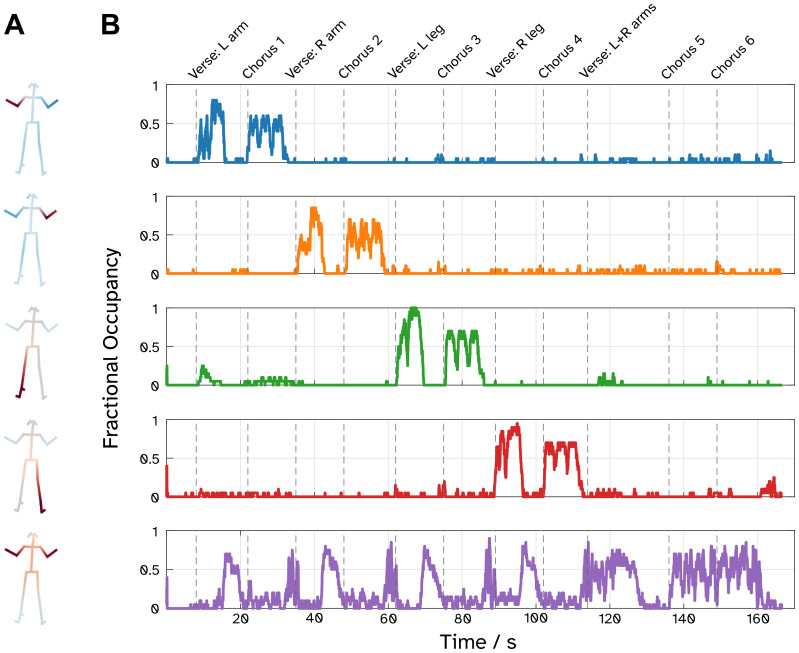
Five telemetry states extracted from the video of the 5 participants dancing to the Hokey Cokey. (A) The heatmaps related to regressing the binarised state timecourses against the key-point velocity data. Deep red areas show the parts of the body represented the most by each state. (B) Session-averaged timecourses for each state, generating a fractional occupancy timeseries, revealing the onset and offset of dominant states over the progression of the dance.

Having established that our video telemetry pipeline works with a traditional, constrained motor paradigm, we extended our investigation to a series of more naturalistic movements during a choreographed dance routine. Of the 25 states extracted from the telemetry, 13 were categorised into 5 meta-states, based on which key-points on the body were employed. These meta-states were used to epoch the MEG data for further analyses. The heatmaps of these meta-states are depicted inFigure 4A. These represent each of the 4 individual limbs and an additional state representing both arms being moved in unison. Individual maps and timeseries of the 25 states can be found in theSupplementary Material.Figure 4Bshows the fractional occupancy of a meta-state across the 15 sessions, which reveals a clear temporal structure emerging from the dance across all subjects and sessions. We see that each of the individual limb states dominates for a verse and chorus and, crucially, the dominant state corresponds to the theme of the verse (*“You put your left arm in”*for verse 1, right arm for verse 2, etc.). We can also observe when a participant switches from one limb to another within a verse. For example, after 16 s when people transition from moving one arm to both at the line*“you do the hokey-cokey”*the dominant state switches from the left arm state (blue line) to the both arms state (purple line).

**Fig. 5.**
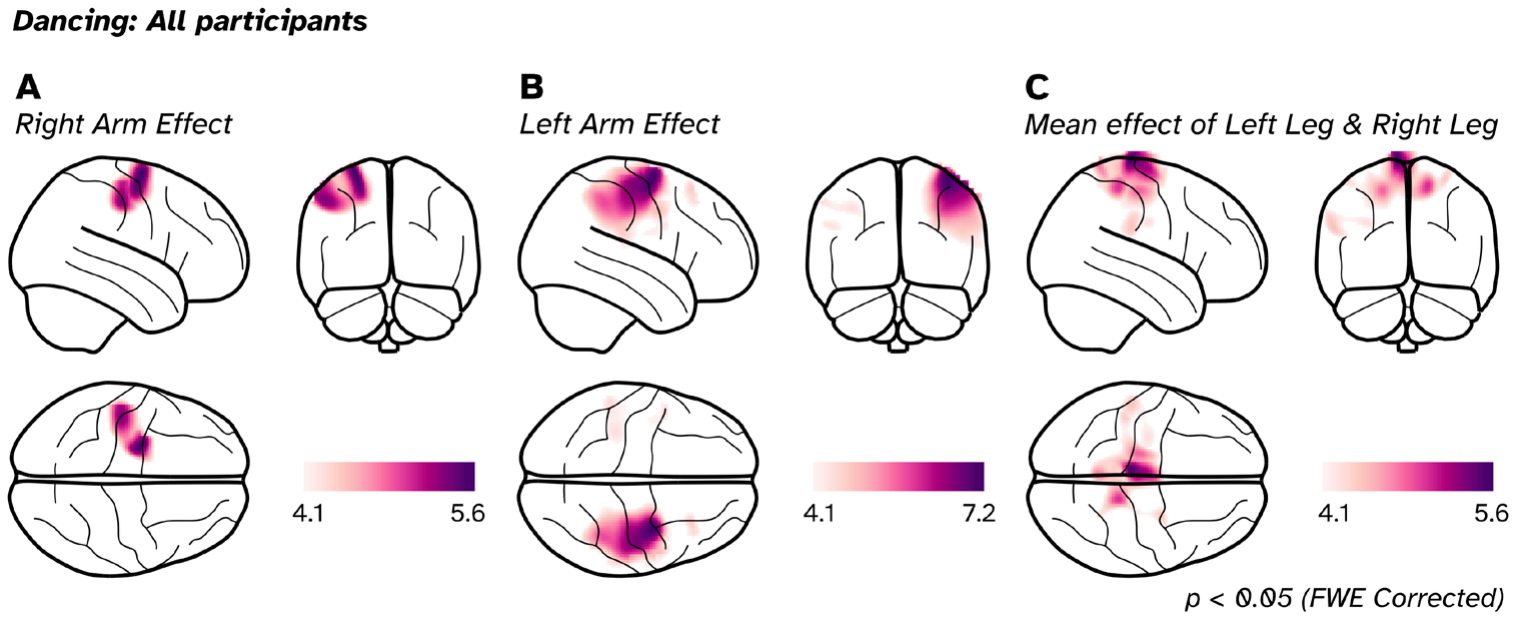
Group activation effects of 8–30 Hz power contrasts between different motor states from participants dancing, shown on glass brains. (A) Right arm effect, derived by contrasting the left arm to both arms. (B) Left arm effect, derived by contrasting right arm to both arms. (C) Combining the left and right leg state contrasts to get the mean effect of both legs reveals reductions in oscillatory power during movement over medial motor areas. For all images, the threshold was set to a T-value where p < 0.05 (FWE corrected).

Activation maps in the 8–30 Hz band, as defined by contrasting different movement states, are depicted inFigure 5. In particular, we focus on three main contrasts of interest. ForFigure 5A and B, we generated paired T-contrasts, where the state for both arms moving (purple state inFig. 4B) was subtracted from one of the single arm states. First, we contrasted when the*left arm*state had more power than the*both arms*state (Fig. 5A) and the resultant paired T-image shows significant activation (p < 0.05, FWE corrected) over the left dorsal sensorimotor areas (associated with right-upper limb movement). To get an activation of the right arm may be counterintuitive, but we note that contrasting both arms to a single arm should result in the activation map of the un-contrasted limb from a set theory perspective (the contrast represents the disjunctive union of the two limbs). Further supporting this idea is the opposite contrast of*both arms*to*right arm*(Fig. 5B), where we see a significant effect in the opposite hemisphere, representing left-arm movement. We note that we also contrasted single arm movements against each other, and these can be found in theSupplementary Material. Finally, we localised activity related to the movement of the legs inFigure 5C. We summed the*left leg*and*right leg*images to make a pooled mean effect of leg activation, finding significant activation in medial areas of the brain corresponding to lower limb movement. In summary, we have been able to extract movement timings from a complex motor paradigm and recover a plausible representation of motor activity in the brain.

## 5 Discussion

Wearable MEG systems (such as those using OPMs) are promising technologies for the future of naturalistic, mobile neuroimaging (Stangl et al., 2023). Work on making wearable MEG systems compatible with immersive virtual environments (Roberts et al., 2019) and ambulatory movement (Holmes et al., 2023b;Mellor et al., 2023;Seymour et al., 2021) is already well underway. Here, we demonstrate that MEG is on its way to being an option for naturalistic studies alongside scalp- and intercranial-EEG (Alexander et al., 2024;Gehrke & Gramann, 2021;Aghajan et al., 2017;Töllner et al., 2017) and fNIRS (Burgess et al., 2022;Oliver et al., 2018). In addition to recording from a mobile imaging modality, the collection, analysis, and fusion of behavioural data during the experiment can return context to more complex neuroimaging data. Here, we combine OP-MEG and video telemetry to show one such path for naturalistic analysis.

We have shown that it is possible to extract experimental timings to process OP-MEG data, entirely from marker-less decomposition of behavioural data, derived with open-source machine-learning approaches applied to videos of participants executing limb movements. We first tested its capabilities in a well-controlled, block-design experiment and found it could recover, and improve on, the experimental timings needed to identify task-based changes in neural activity. We then applied this approach to a dancing paradigm, where, after some basic choreography instruction to the participants, all movement was based on the interpretation and timing of each individual. From these video recordings, we derived states associated with movements of different limbs, which were mapped onto plausible representations of the limbs along the sensorimotor cortex. Our approach is particularly applicable for naturalistic studies, where consistent timings across participants are not guaranteed. By decoding these motor states from the behavioural data, we recover the subject-specific timings and factor in what could be previously described as ‘noise’ in our experimental design. This had been identified as one of the key challenges to developing successful mobile, naturalistic neuroimaging studies (Stangl et al., 2023).

Using regular (visual spectrum) video data to analyse movement with neural-network-based pose estimators (Bazarevsky et al., 2020;Cao et al., 2021;Mathis et al., 2018) demonstrates that one can forgo the need for additional retro-reflective markers on the body. The use of pose estimators as a valid method to supplement or replace more extensive motion capture systems is currently an active area of investigation (Bae et al., 2024;Friedrich et al., 2024;Kosourikhina et al., 2022;Needham et al., 2021), with initial findings confirming similar performance between systems. Using pose estimators on visible light data has an additional benefit for the family of OPMs we use, such that it removes a source of infra-red light from traditional motion capture, that can interfere with the operation of our OPMs. The use of HMMs to partition motion telemetry data is not uncommon (Agrahri et al., 2022;Buderman et al., 2021;Conners et al., 2021) as they are able to exploit the temporally-rich nature of the multivariate motion data. We note that HMMs are also very popular in neuroimaging analysis—indeed, we made use of an HMM toolbox primarily designed to accommodate neuroimaging data here (Vidaurre et al., 2016).

Our results clustered into locations of the upper limb movements (dorsal motor cortex) and lower limb areas (medial motor cortex) that conform with the functional neuroanatomy of the sensorimotor cortices (Gordon et al., 2023;Jensen et al., 2022). This separation was clear in the block-design motor experiment, where participants were seated and it was easier for them to move a limb in isolation. For the dancing paradigm, participants were moving their whole bodies during each dance move, and never just moved one limb in isolation. This explains, for example, why there was activation within lower limb areas only when contrasting the leg states together rather than subtracting from each other, as muscles in both legs are activated when executing a swing movement with one leg. This investigation provides a proof-of-principle of the potential of wearable MEG to facilitate functional neuroimaging without highly controlled behavioural tasks.

We note that our results were based on the modulation of induced oscillatory power, where precise time- and phase-locking to actions or stimuli are not essential to reveal the movement-related dynamics in the telemetry. Optical motion capture methods, whether marker-less pose estimators or tracking of retro-reflective markers, are essentially limited to the performance of the cameras used. These cameras run at approximately 200 frames per second for most practical applications. This temporal precision (~5 ms) is likely sub-optimal for most average evoked response measures. Conventional methods to detect movement from a small area of the body (e.g., from an electromyogram or an inertial measurement unit) offer a temporal precision currently unmatched and are suitable for evoked response measures. However, this temporal precision comes at the cost of a lack of spatial coverage. That is, there is an experimental trade-off between temporal precision and coverage.

Tracking whole-body subject motion also has clinical value. Pose-based behavioural data has been shown to be beneficial in assessing Parkinson’s disease (Kaneko et al., 2024;Sabo et al., 2022;Roth et al., 2021), the progress of Friedreich’s ataxia (Kadirvelu et al., 2023), detecting non-verbal behaviour in Autistic Spectrum Disorder (Kojovic et al., 2021), and predicting cerebral dysfunction in infants (Gleason et al., 2024). Furthermore, combining pose-estimation with a flexible neuroimaging system such as OP-MEG (or EEG) promises to improve our understanding of these conditions in ways that were previously unavailable. There are direct clinical pathways where this could offer immediate benefit, such as in paediatric cases. For example, cases of refectory (drug resistant) epilepsy where surgical intervention may require careful localisation of the eloquent cortex with functional neuroimaging, to ensure that key faculties are not impaired when removing the seizure onset zone. This is typically assessed with functional MRI (Al-Arfaj et al., 2023;Barras et al., 2016), but success of the mapping is limited to the performance of the patient, and their tolerance of the MR system. For young children with epilepsy, this effect may be compounded (Yerys et al., 2009). Solutions to this could include trying to map the motor areas in the resting state (Krishnamurthy et al., 2022), but there may be a more naturalistic alternative. Children playing when undergoing video-M/EEG telemetry sessions for seizure monitoring will enter states of movement and periods of rest naturally. If the video footage was processed to demarcate these periods, it is entirely feasible that these motor maps (and, indeed, language or other key regions of eloquent cortex) could be derived*for free*out of long recordings where the primary goal is to locate ictal/interictal activity. Using play in neuroimaging may also be favourable for other populations, such as investigating the progress of motor development in children with Autistic Spectrum Disorder (An et al., 2021;Wilson et al., 2018), where a flexible imaging system which can adapt to the child, while simultaneously tracking movement should lead to more successful recording sessions than a large unadaptable system.

One technical aspect of the OP-MEG acquisition is the lack of additional active shielding applied to our OPM recordings. Our magnetically shielded room provides adequate shielding in the centre of the room to provide a low enough field to keep the sensors in their operational range (up to 4.5 nT from their initialisation point). However, we did not account for cross-axis projection errors (CAPE;Borna et al., 2022), which become apparent in recordings when the background field deviates by approximately 1 nT or more from the sensor’s zero-field point. Cross-axis projection errors result in additional asymmetrical non-linearities in the field-to-voltage response of the sensors (Schofield et al., 2023), which, in turn, has an impact on source localisation performance (Borna et al., 2022). In our block-designed motor experiment, the largest field experienced by any sensor was 2.1 nT from its zero-point. For the dancing data, the largest fields measured for each of the 5 subjects were 1.5, 0.6, 1.2, 2.6, and 3.5 nT from their zero-point. While the data presented here will contain CAPE effects, their effect is small and the interference reduction methods applied here are robust to the non-linearities introduced by CAPE (Tierney et al., 2022). Methods which counteract field changes experienced by the sensors during ambulatory motion, whether through dedicated nulling coils built into the shielded room (Holmes et al., 2023b) or by using a dynamic closed-loop system built directly into the sensors (Lee et al., 2014;Mellor et al., 2023;Nardelli et al., 2019;Robinson et al., 2022), could be employed for future studies to control for CAPE-related non-linearities.

To conclude, our work provides additional support for the exciting opportunity of OP-MEG for studying the neural basis of complex motor functions, but also spatial navigation, memory, and social interactions in realistic and ecologically valid situations, in both health and disease.

## Supplementary Material

Supplementary Material

## Data Availability

The MATLAB scripts used to analyse the data are available athttps://github.com/georgeoneill/study-videomeg. Openpose 1.7.0 is available to download fromhttps://github.com/CMU-Perceptual-Computing-Lab/openpose. The data, downsampled to 2 kHz (with anti-aliasing applied), are available to download from Zenodo athttps://doi.org/10.5281/zenodo.8139849. Openpose telemetry data from the video are included. To conform with data protection regulations, we cannot share the original video on which the pose inference was performed.
